# MiR-139-5p inhibits migration and invasion of colorectal cancer by downregulating AMFR and NOTCH1

**DOI:** 10.1007/s13238-014-0093-5

**Published:** 2014-08-23

**Authors:** Mingxu Song, Yuan Yin, Jiwei Zhang, Binbin Zhang, Zehua Bian, Chao Quan, Leyuan Zhou, Yaling Hu, Qifeng Wang, Shujuan Ni, Bojian Fei, Weili Wang, Xiang Du, Dong Hua, Zhaohui Huang

**Affiliations:** 1 Wuxi Oncology Institute, the Affiliated Hospital of Jiangnan University, Wuxi, 214062 China; 2 Department of Surgical Oncology, the Affiliated Hospital of Jiangnan University, Wuxi, 214062 China; 3 Department of Radiation Oncology, the Affiliated Hospital of Jiangnan University, Wuxi, 214062 China; 4 Department of Pathology, Fudan University Shanghai Cancer Center, Shanghai, 200032 China

**Keywords:** miR-139-5p, AMFR, NOTCH1, colorectal cancer, metastasis, prognosis

## Abstract

MicroRNAs (miRNAs) that exert function by posttranscriptional suppression have recently brought insight in our understanding of the role of non-protein-coding RNAs in carcinogenesis and metastasis. In this study, we described the function and molecular mechanism of miR-139-5p in colorectal cancer (CRC) and its potential clinical application in CRC. We found that miR-139-5p was significantly downregulated in 73.8% CRC samples compared with adjacent noncancerous tissues (NCTs), and decreased miR-139-5p was associated with poor prognosis. Functional analyses demonstrated that ectopic expression of miR-139-5p suppressed CRC cell migration and invasion *in vitro* and metastasis *in vivo.* Mechanistic investigations revealed that miR-139-5p suppress CRC cell invasion and metastasis by targeting AMFR and NOTCH1. Knockdown of the two genes phenocopied the inhibitory effect of miR-139-5p on CRC metastasis. Furthermore, the protein levels of the two genes were upregulated in CRC samples compared with NCTs, and inversely correlated with the miR-139-5p expression. Increased NOTCH1 protein expression was correlated with poor prognosis of CRC patients. Together, our data indicate that miR-139-5p is a potential tumor suppressor and prognostic factor for CRC, and targeting miR-139-5p may repress the metastasis of CRC and improve survival.

**Electronic supplementary material:**

The online version of this article (doi:10.1007/s13238-014-0093-5) contains supplementary material, which is available to authorized users.

## INTRODUCTION

MicroRNAs (miRNAs) are small endogenous non-coding RNAs that regulate the expression of target genes at the post-transcriptional level through sequence-specific binding to the 3′ untranslated region (3′UTR) of their specific mRNAs, resulting in translational inhibition even destruction of the target mRNAs (Bartel 2004). MiRNAs play vital roles in tumorigenesis by acting as tumor suppressors, such as let-7, miR-143, and miR-200 (Chen et al. 2009; Li et al. 2009), or oncogenes, such as miR-21, miR-155, and miR-95 (Asangani et al. 2008; Huang et al. 2011; Hwang et al. 2014). Deregulation of miRNAs is a common event in human tumors and involves in various pathological processes, possessing with either oncogenicity or promotion of metastasis. Though recent researches of miRNAs have brought mind-blowing insight into our knowledge of human cancers, there are still largely unknown details that need to be explored further.

Colorectal cancer (CRC) is one of the most commonly diagnosed cancers in both genders worldwide. Over 1.2 million new cancer cases occur annually, resulting in greater than 600,000 deaths each year (Jemal et al. 2011). Although some advances are achieved in the diagnosis and treatment, the disease is very often diagnosed at an advanced stage with tumor metastasis, making it starving for the discovery of new metastasis relevant molecules that could help to improve the diagnosis and prognosis.

Many studies have shown that certain miRNAs play important roles in various processes of the metastatic cascade, such as cell adhesion, migration, invasion, angiogenesis, and epithelial-mesenchymal transition (EMT) (Hurst et al. 2009; Nicoloso et al. 2009). However, detailed mechanisms that contribute to metastasis process remain poorly understood. Our previous data revealed that miR-139-5p was downregulated in CRC (Huang et al. 2011). Interestingly, decreased expression of miR-139-5p has also been found in several other tumors, including breast cancer, gastric cancer, hepatocellular carcinoma, glioblastoma, and laryngeal squamous carcinoma (Guo et al. 2009; Gu et al. 2013; Krishnan et al. 2013; Li et al. 2013; Liu et al. 2013). Recent data suggested that miR-139 may inhibit CRC growth and/or metastasis by targeting different genes (Guo et al. 2012; Shen et al. 2012; Zhang et al. 2014). However, their results were contradictory. The accurate role and clinical value of miR-139-5p in CRC remain to be unclear. In this study, we confirmed the downregulation of miR-139-5p in an expanded CRC cohort, which correlated with poor survival of CRC patients, and revealed that miR-139-5p could inhibit CRC invasion and metastasis through directly targeting AMFR and NOTCH1 in CRC.

## RESULTS

### Decreased miR-139-5p expression is associated with poor overall survival in CRC

**Figure 1:**
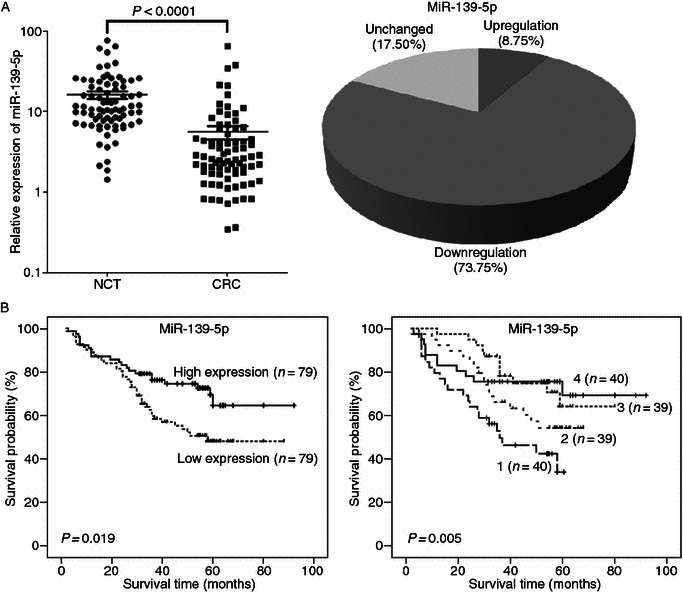
**miR-139-5p is frequently downregulated and associated with poor overall survival in CRC**. (A) MiR-139-5p expression was detected by quantitative reverse transcription polymerase chain reaction (qRT-PCR) in 80 paired CRC and adjacent noncancerous tissues (NCTs). MiR-139-5p expression was markedly downregulated in tumor tissues compared with the corresponding NCTs (U6 small nuclear RNA was used as an internal control). (B) Overall survival analysis based on the expression level of miR-139-5p. MiR-139-5p expression was examined in 158 CRC tissues, and these cases were divided into two groups (high or low) or four groups (1–4) based on their miR-139-5p levels in tumors. MiR-139-5p expression was positively correlated with the overall survival

Our data of miRNA expression profile have demonstrated that miR-139-5p is downregulated in CRCs compared to NCTs (Huang et al. 2011). To further verify the result, the expression level of miR-139-5p was examined using qRT-PCR in an expanded cohort of 158 CRCs, including 80 tumors with paired NCTs. In accordance with the microarray data, the results showed that miR-139-5p was downregulated more than 2-fold in 73.8% of CRCs compared with NCTs (*P* < 0.0001, Fig. 1A). Survival analysis showed that high miR-139-5p expression was associated with prolonged overall survival of CRC patients (high: the 25% highest, low: the 25% lowest) (*P* < 0.05, Fig. 1B). After adjustment for age, gender, tumor size, pathologic stage, and grading, a Cox multivariate analysis indicated that miR-139-5p expression is an independent prognostic factor for CRC (adjusted HR = 2.681, 95% CI = 1.532–4.691, *P* = 0.001, Table S1). No significant relationship was found between miR-139-5p expression in CRC and tumor size, location, stage, and grading (*P* > 0.05).

### Overexpression of miR-139-5p inhibits CRC cell migration and invasion *in vitro* and metastasis *in vivo*

**Figure 2:**
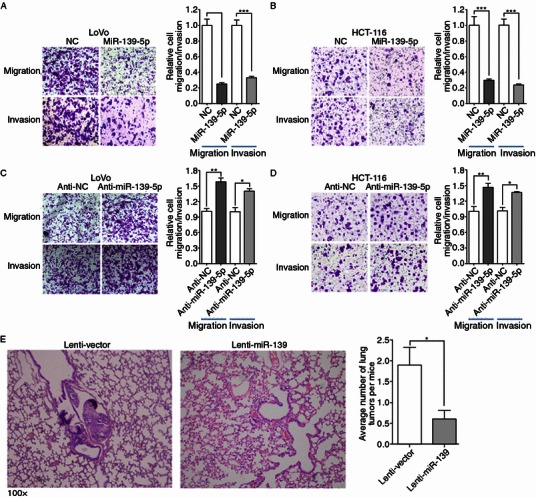
**MiR-139-5p inhibits CRC cell migration and invasion*in vitro*and*in vivo***. (A and B) The overexpression of miR-139-5p inhibits the migration and invasion of LoVo and HCT-116 cells. (C and D) The silencing of miR-139-5p enhanced the migration and invasion of LoVo and HCT-116 cells. (E) The effect of miR-139 on tumor metastasis in a lung metastasis model of nude mouse. LoVo cells stably expressing miR-139 or the control (2 × 10^6^) were injected into the caudal vein of each nude mouse. The ectopic expression of miR-139 significantly reduced the number of lung metastases (*P < 0.05)

Now that miR-139-5p was downregulated in CRC and associated with poor prognosis, we conclude that miR-139-5p may have an inhibitory role on CRC cell proliferation and/or invasion. To determine whether miR-139-5p could regulate the metastasis ability of CRC, we first examined the effect of miR-139-5p on CRC cell mobility. The results showed that miR-139-5p overexpression can suppress the cell migration and invasion (Fig. 2A and 2B), whereas silencing of miR-139-5p promotes cell migration and invasion (Fig. 2C and 2D). To identify the impact of miR-139-5p on *in vivo* metastasis of CRC cells, LoVo cells stably expressing miR-139-5p or the vector control were injected into the caudal vein of athymic BALB/c nude mice. Ectopic expression of miR-139-5p significantly reduced the number of lung metastasis sites (Fig. 2E and Table S2), further validating that the invasive behaviour of CRC could be suppressed by miR-139-5p. In contrast, no significant effect of miR-139-5p on cell proliferation was observed based on the *in vitro* and *in vivo* analyses (Fig. S1). Because pri-miR-139 could produce both miR-139*-*3p and miR-139-5p, we checked the expression and function of miR-139*-*3p in CRC cells. The results showed that the expression of miR-139*-*3p in CRC cells was much lower than that of miR-139-5p (Fig. S2A and S2B), and overexpression of miR-139*-*3p showed no significant effect on CRC cell growth and migration (data not shown). Collectively, these data indicate that miR-139-5p was able to suppress CRC migration and invasion.

### AMFR and NOTCH1 are the direct target genes of miR-139-5p in CRC

**Figure 3:**
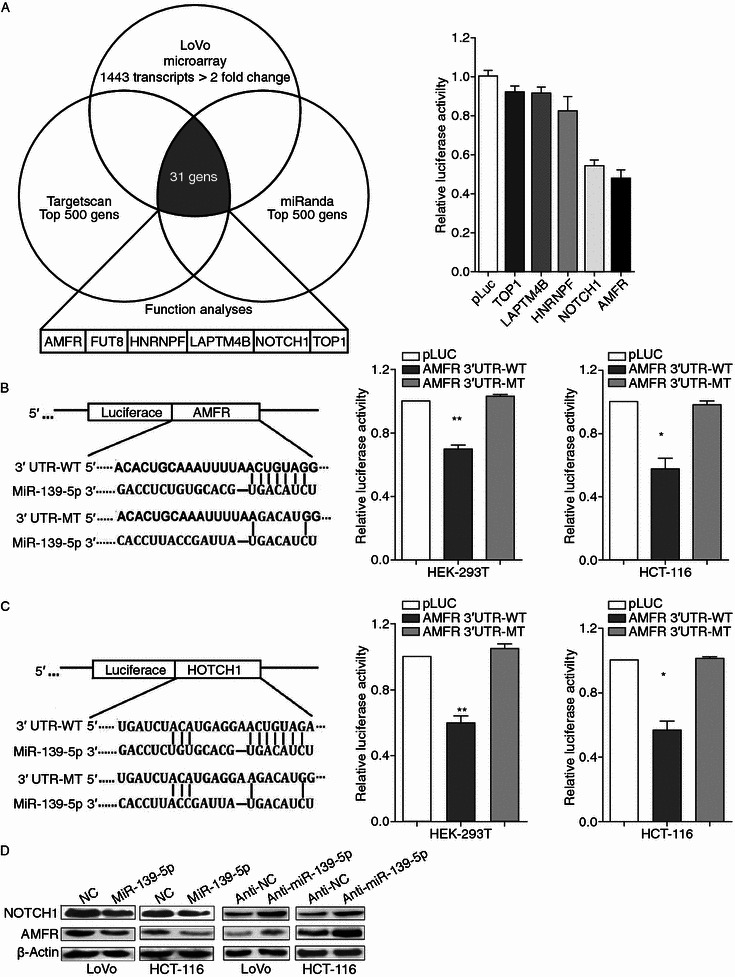
**AMFR and NOTCH1 are the direct target genes of miR-139-5p**. (A) Initial screening of miR-139-5p target genes using a microarray assay, bioinformatics predictions and the luciferase reporter assay. Five downregulated genes (AMFR, NOTCH1, HNRNPF, TOP1, and LAPTM4B) were selected from the 31 genes in the initial screening based on the functional analysis of these genes, and their 3′UTRs were assessed using the luciferase reporter assay. (B and C) Analyses of the luciferase activity of the luciferase reporter plasmids containing either wild-type (WT) or mutant-type (MT) 3′UTRs of AMFR and NOTCH1 in HEK-293T and HCT-116 cells. A mutation was generated in the site complementary to the miR-139-5p seed region of the 3′UTR of AMFR or NOTCH1, as indicated. (D) The protein levels of AMFR and NOTCH1 were determined by Western blot assays using LoVo and HCT-116 cells transfected with miR-139-5p mimic, miR-139-5p inhibitor (anti-miR-139-5p) or their corresponding negative control (NC or anti-NC). Beta-actin protein was used as an internal control

To investigate the mechanism by which miR-139-5p exerts anti-metastasis function in CRC, we searched for the potential targets of miR-139-5p using bioinformatics algorithms (TargetScan and miRanda) and a microarray assay. Genomic-wide expression profiling was performed in NC- and miR-139-5p-transfected LoVo cells using a microarray. A total of 1443 downregulated transcripts (>2-fold change) were identified in miR-139-5p-transfected cells compared with the control (Table S3). Among the top 500 targets of miR-139-5p predicted by the two algorithms, 31 genes were downregulated in the miR-139-5p-transfected cells. Then, 5 potential target genes with tumor-promoting function (AMFR, NOTCH1, HNRNPF, TOP1, and LAPTM4B) were selected from the 31 genes and their 3′UTRs containing the complementary binding sites of miR-139-5p were cloned into a luciferase reporter vector to evaluate the influence of miR-139-5p on the expression of a reporter gene using a luciferase assay (Fig. 3A). The expression of the reporter gene in the recombinant plasmids containing AMFR and NOTCH1 3′UTRs were significantly repressed by miR-139-5p. To further confirm that AMFR and NOTCH1 are direct targets of miR-139-5p in CRC, we mutated the predicted binding sites of miR-139-5p in the 3′UTRs of AMFR and NOTCH1 that are conserved among mammals, and found that the mutant 3′UTRs were completely refractory to miR-139-5p-mediated luciferase reporter repression in HEK-293T and HCT-116 cells (Figs. 3B, 3C and S3). In line with these results, the endogenous AMFR and NOTCH1 protein levels were also decreased in miR-139-5p-overexpressing CRC cells and could be restored in miR-139-5p-depleted cells (Fig. 3D).

### The protein expression of AMFR and NOTCH1 is upregulated in CRC and negatively correlated with miR-139-5p expression

**Figure 4:**
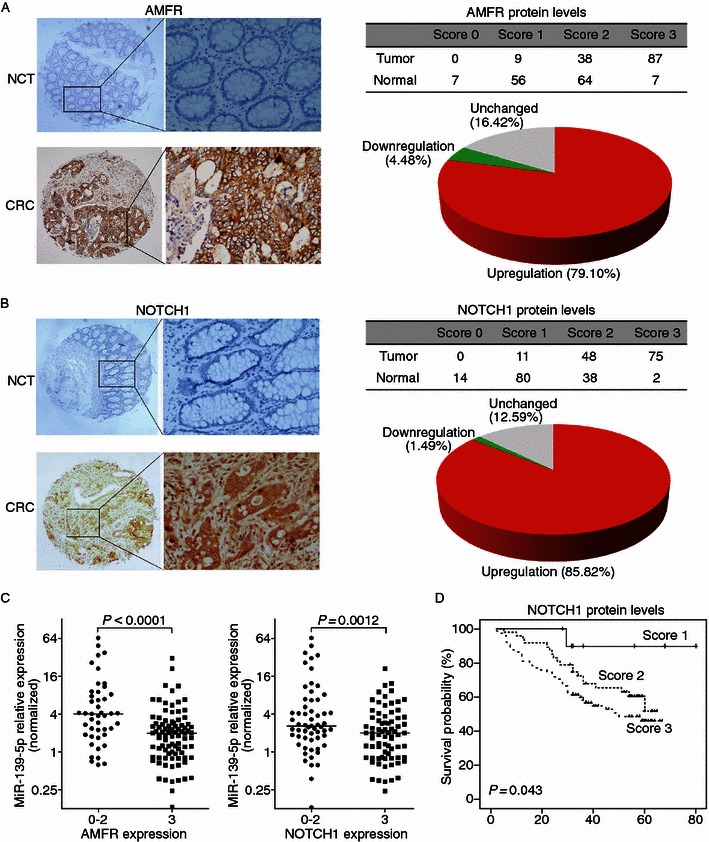
**The protein expression of AMFR and NOTCH1 was upregulated in CRC and negatively correlated with miR-139-5p expression**. (A and B) Immunohistochemical staining of AMFR and NOTCH1 in CRC tissues and corresponding noncancerous tissues (NCTs). Brown cytoplasmic AMFR/NOTCH1 staining was strong in CRC tissues but nearly absent in the normal epithelia. AMFR and NOTCH1 protein expression were frequently increased in the tumor tissues compared with the matched NCTs. (C) The expression levels of AMFR and NOTCH1 negatively correlated with the miR-139-5p levels in the CRC tissues. (D) Survival analysis based on the expression levels of NOTCH1 protein. The groups were ranked according to the NOTCH1 staining intensity

To further evaluate the relationship between miR-139-5p and AMFR/NOTCH1, we detected their expression in paired CRC and NCT tissues using qRT-PCR and immunohistochemistry, respectively. Brown cytoplasmic AMFR and NOTCH1 staining was strong in CRC tissues but nearly absent in the normal epithelia (Fig. 4A, 4B and S4). Of the 134 cases, 106 tumors showed increased AMFR expression and 115 tumors showed increased NOTCH1 expression compared with the paired NCTs. The protein expression of AMFR and NOTCH in tumor tissues inversely correlated with the miR-139-5p levels (AMFR: Spearman *r* = −0.352, *P* = 0.0001; NOTCH1: Spearman *r* = −0.184, *P* = 0.033, Fig. 4C). Interestingly, a positive correlation was observed between the protein expression of AMFR and NOTCH1 in CRC (Spearman *r* = 0.986, *P* < 0.0001), suggesting that they may be regulated by same mechanisms. Taken together, these data suggest that miR-139-5p inactivation may result in the increased expression of AMFR and NOTCH1 in human CRC.

Survival analyses revealed that increased NOTCH1 protein levels were associated with shorter survival time (*P* < 0.05, Figs. 4D and S5). After adjusting for age, gender, tumor size, tumor grading, and tumor stage, multivariate analyses showed that NOTCH1 expression was an independent risk factor for survival. Tumors with higher NOTCH1 expression (score 3) presented a higher risk of death (HR = 1.984, 95% CI = 1.238–3.180, *P* = 0.004). No significant association was found between AMFR expression and survival.

### MiR-139-5p inhibits tumor invasion via directly targeting AMFR and NOTCH1 in CRC

**Figure 5:**
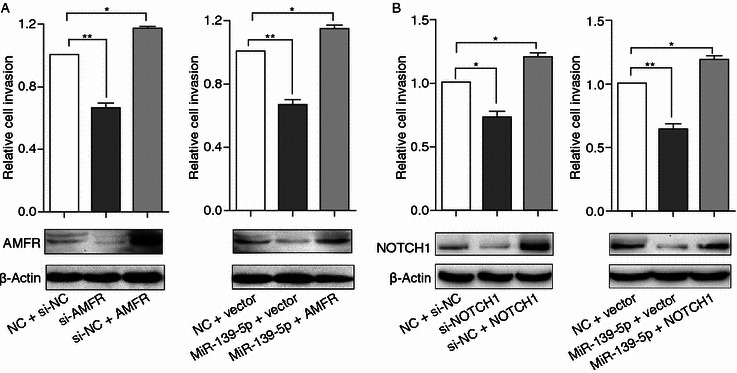
**MiR-139-5p inhibits CRC invasion via directly targeting AMFR and NOTCH1 in CRC**. (A and B) The knockdown of AMFR or NOTCH1 by siRNA significantly repressed CRC cell invasion, which phenocopied the function of miR-139-5p, whereas the overexpression of AMFR or NOTCH1 (ORF without 3′UTR) markedly promoted cell invasion and counteracted miR-139-5p-induced invasion inhibition in LoVo cells

To examine the functional effect of AMFR and NOTCH1 in miR-139-5p-induced metastasis inhibition in CRC cells, we inhibited AMFR and NOTCH1 expression with siRNA (Fig. S6) and revealed that AMFR- and NOTCH1-depleted LoVo cells showed decreased invasion, which phenocopied the invasion-inhibiting effect of miR-139-5p. In contrast, the ectopic overexpression of AMFR and NOTCH1 using plasmids of AMFR and NOTCH1 (ICN) ORF promoted cell invasion, which could not be repressed by the overexpression of miR-139-5p (Fig. 5A and 5B). Taken together, these results proved that miR-139-5p inhibits CRC invasion through AMFR and NOTCH1.

## DISCUSSION

Nowadays more than 2000 miRNAs have been identified in human, which participate in keeping the balance of gene regulating networks and regulate more than a third of human genes at the post-transcription level (Esquela-Kerscher and Slack 2006). The disruption of microRNAs may be crucial in numerous cellular processes, particularly in oncogenesis and metastasis (Manikandan et al. 2008; Bouyssou et al. 2014). MiR-139-5p, one of the most significant downregulated miRNA in CRC in our previous data (Huang et al. 2011), have been identified as relating to the development of several tumor types (Bao et al. 2011; Wong et al. 2011; Krishnan et al. 2013; Liu et al. 2013), including CRC (Guo et al. 2012; Shen et al. 2012; Zhang et al. 2014). Here, we identified miR-139-5p as a metastasis inhibitor through directly targeting AMFR and NOTCH1 in CRC.

MiR-139 shows extensive function in human tumorigenesis and development. Wong first reported that miR-139 suppresses metastasis and progression of hepatocellular carcinoma by downregulating Rho-kinase 2 (Wong et al. 2011). Bao et al. revealed that HER2 and CD44 can induce histone deacetylation in the promoter region of the miR-139 gene, resulting decreased miR-139 expression and increased expression of its target CXCR4 in gastric cancer (Bao et al. 2011). Subsequent reports showed that miR-139-5p suppress the invasive capability of breast cancer (Krishnan et al. 2013) and esophageal carcinoma cells (Liu et al. 2013). These reports indicate the common key role of miR-139-5p in cancer metastasis. In this study, we confirmed the downregulation of miR-139-5p in CRC in an expanded cohort of CRC (Fig. 1A). Further functional analyses demonstrated that the overexpression of miR-139-5p can significantly suppress cell migration and invasion *in vitro* and metastasis *in vivo* (Fig. 2). These results were consistent with the results of Shen et al. and Zhang et al. (Shen et al. 2012; Zhang et al. 2014). However, Guo et al. and Zhang et al. reported that miR-139-5p could inhibit CRC cell growth *in vitro* (Guo et al. 2012; Zhang et al. 2014), which was not observed in the study of Shen et al. (Shen et al. 2012). We also did not observe the growth-inhibiting function of miR-139-5p using *in vitro* and *in vivo* assays (Fig. S1). It may be due to different methodology or cell lines used in different studies. These conclusions should be validated under the same conditions in the future work.

AMFR, located on human chromosome 16q^12.2^, known as gp78, is a ubiquitin E3 ligase involved in the degradation of proteins through the endoplasmic reticulum pathway (Fang et al. 2001). AMFR has the pluripotency activated by AMF involving cells adhesion, motility, and angiogenesis (Yanagawa et al. 2004; Chiu et al. 2008). AMF stimulates AMFR in an autocrine manner and results in signalling cascades relying on PKC and activates Rho-like GTPase, RhoA, and Rac1, which enhance cell motility (Kanbe et al. 1994; Wang et al. 2010). Several groups have shown that AMFR upregulation correlates with more advanced tumor stage and poor survival in stomach, lung, and breast cancer (Taniguchi et al. 1998; Kawanishi et al. 2000; Jiang et al. 2006), and appears to be a strong independent predictor for recurrence in CRC (Nakamori et al. 1994). Although recent data suggested that AMFR may be associated with tumorigenesis and development, the detailed function and mechanisms of AMFR remain unknown (Wang et al. 2010; Kho et al. 2013). In this study, we showed, for the first time, that AMFR was regulated by miR-139-5p and could promote CRC invasion, and the expression of AMFR protein was negatively correlated with miR-139-5p in CRC tissues, suggesting that the increasing activity of AMFR was due to the inactivation of miR-139-5p in CRC (Figs. 3 and 4). The detailed mechanism by which AMFR promotes CRC cell metastasis should be investigated in future study.

NOTCH1 is another target gene of miR-139-5p identified in CRC (Zhang et al. 2014). Four NOTCH receptors (NOTCH1–4) and five ligands (Jagged-1, 2, Delta-1, 3, 4) have been described in mammals (Miele et al. 2006). Dysregulated activity of NOTCH signaling is frequently observed in human cancers and a lot of studies have suggested an involvement of NOTCH signaling in cancer angiogenesis and metastasis (Hellstrom et al. 2007; Hu et al. 2012). NOTCH1 is found upregulated in CRC and correlates with tumor proliferation, differentiation, lymphatic metastases, and survival time (Reedijk et al. 2008; Chu et al. 2010; Hristova et al. 2013). We observed that NOTCH1 was directly regulated by miR-139-5p at the post-transcriptional level and promoted CRC invasion. And our data showed that the expression of NOTCH1 was significantly upregulated in CRC and negatively correlated with miR-139-5p and survival (Figs. 3 and 4). In line with our results, the metastasis-prompting function of NOTCH1 has been reported in breast cancer, prostate cancer, esophageal cancer, and melanoma (Wang et al. 2010; Hu et al. 2012). In addition to miR-139-5p, other miRNAs (miR-34a, miR-144) were also reported to inhibit NOTCH1 in CRC (Sureban et al. 2011; Bu et al. 2013), indicating that there is a significant cross-talk between NOTCH and miRNAs (Mo et al. 2013).

In conclusion, we determined that miR-139-5p is downregulated in CRC and appears to be a prognostic factor for CRC, and miR-139-5p inhibits CRC invasion and metastasis by targeting AMFR and NOTCH1. These data suggest that restoration of miR-139-5p may be a promising therapeutic strategy for anti-metastasis treatment of CRC.

## MATERIALS AND METHODS

### Human tissues and cell lines

A total of 158 human primary CRC tissues and their paired adjacent noncancerous tissues (NCTs) were collected between 2005 and 2008 at Affiliated Hospital of Jiangnan University and Fudan University Shanghai Cancer Center. The detailed clinical information for the CRC patients is included in the Supplementary Data (Table S4). All of the materials of patients were obtained with informed consent, and this project was approved by the Clinical Research Ethics Committees of the Affiliated Hospital of Jiangnan University and Fudan University Shanghai Cancer Center. HEK-293T cell line and human CRC cell lines, including HCT-8, HCT-116, LoVo, and SW480, were purchased from American Type Culture Collection (ATCC). HCT-8, HCT-116, and LoVo cells were cultured in RPMI-1640, McCoy’s 5a, and F12-K medium, respectively. HEK-293T and SW480 cells were maintained in DMEM and Leibovitz’s L-15 medium, respectively. All of the media (Hyclone, USA) were supplemented with 10% fetal bovine serum (Gibco, USA), plus penicillin (100 U/mL) and streptomycin (100 U/mL). The cells were incubated under the conditions recommended by ATCC.

### RNA extraction and quantitative real-time RT-PCR

Total RNA was extracted using the TRIzol reagent (Invitrogen, USA) according to the manufacturer’s instructions. The concentration of RNA was measured using a NanoDrop ND-1000 instrument (NanoDrop, USA). cDNA was synthesised using the PrimeScript RT reagent kit (TaKaRa, Japan). qPCR analyses were conducted to quantitate mRNA relative expression using SYBR Premix Ex Taq (TaKaRa, Japan) with beta-actin as an internal control. TaqMan microRNA assays (Applied Biosystems, USA) were used to determine miRNA expression levels (Applied Biosystems, USA) with U6 small nuclear RNA as an internal control. The results of qPCR were defined from the threshold cycle (Ct), and relative expression levels were calculated by using the 2^-△△Ct^ method (Livak and Schmittgen 2001). PCR was performed using ABI 7300HT and ABI Vii7 instrument (Applied Biosystems, USA). The primers used for PCR analysis were listed in the Table S5.

### Vector constructs

The human pri-miR-139 sequence was amplified from normal human genomic DNA by nested PCR using PrimerSTAR Premix (TaKaRa, Japan). The sequence was then cloned into the lentivirus expression vector pWPXL (Clontech, USA) to generate pWPXL-miR-139. The predicted binding sites in the 3′UTRs of the potential target genes of miR-139-5p were amplified by nested PCR and cloned into the region directly downstream of a CMV promoter-driven firefly luciferase cassette in the pcDNA3.0 vector (pLUC). The mutant 3′UTRs of AMFR and NOTCH1, which carry the mutated sequence in the complementary site of the seed region of miR-139-5p, were generated from their 3′UTR-WT plasmids by overlap-extension PCR. The sequences of the wild-type and mutant 3′UTRs were confirmed by DNA sequencing. The open reading frame (ORF) of AMFR was amplified via nested PCR and cloned into the pWPXL vector. The intracellular domain of the NOTCH1 (ICN) plasmid pSIN-EF2-ICN was kindly provided by Prof. Lei Dong (State Key Laboratory of Pharmaceutical Biotechnology, School of Life Sciences, Nanjing University). The primers and endonuclease sites used for the vector constructs are shown in the Supplementary Data (Table S5).

### Oligonucleotide transfection

MiR-139-5p mimics and inhibitors (anti-miR-139-5p, chemically modified antisense oligonucleotides designed to specifically target mature miR-139-5p), as well as their corresponding negative control (NC), were synthesised at Ribobio (Guangzhou, China). AMFR and NOTCH1 small interfering RNA (si-AMFR target sequence: 5′-GTACATACATCTCCTACAA-3′; siNOTCH1: 5′-GUCCAGGAAACAACUGCAATT-3′) were purchased from GenePharma (Shanghai, China). Oligonucleotide transfection was performed using Lipofectamine 2000 reagents (Invitrogen, USA) according to the manufacturer’s instructions.

### Lentivirus production and transduction

Lentivirus production and transduction was performed as described previously (Huang et al. 2011).

### Cell migration and invasion assay

For the migration assays, 1 × 10^5^ HCT-116 or 5 × 10^4^ LoVo cells (stably expressed miR-139 or vector control) in 200 μL serum-free medium were placed into the top chamber of each insert (BD Biosciences, NJ), and 800 μL medium supplemented with 10% fetal bovine serum into under chamber, After 24 (HCT-116) or 8 (LoVo) h of incubation at 37°C, cells adhering to the lower membrane were stained with 0.1% crystal violet containing 20% methanol, imaged, and counted using an IX71 inverted microscope (Olympus, Japan). For the cell invasion assay, the polycarbonate membranes of the upper compartment of the chambers were pre-coated with a matrix gel.

### Cell proliferation assay

Cell proliferation was quantified using the Cell Counting Kit-8 (CCK8; Dojindo Laboratories, Japan) according to the manufacturer’s Manual as described previously (Huang et al. 2011).

### Tumor formation assay in a nude mouse model

Athymic male BALB/c nude mice were feeding under specific pathogen-free conditions in the Experimental Animal Department of Fudan University. The assay protocols were approved by the Shanghai Medical Experimental Animal Care Commission. For the *in vivo* tumor formation assays, 4 × 10^6^ LoVo cells stably expressing miR-139 or the vector control were subcutaneously injected into either flank of the same athymic male BALB/c nude mouse at 5 weeks of age (*n* = 6 for each group). After transplantation, the growth of the tumors was assessed twice a week. The mice were sacrificed after a period of 4–6 weeks, and the weight of subcutaneous tumors were calculated. For the *in vivo* metastasis assays, 2 × 10^6^ LoVo cells stably expressing miR-139 or the control vector were suspended in 100 μL of DMEM and were injected into the caudal vein of each nude mouse (*n* = 10 for each group). The nude mice were maintained under specific pathogen-free conditions in the Experimental Animal Department of Fudan University. The mice were sacrificed after a period of 5 weeks.

### Microarray analysis

Expression profiling was performed using an Agilent human whole genome oligo microarray chip (4 × 44 K) (Agilent, USA). A total of 5 × 10^5^ LoVo cells were seeded in 6-cm^2^ tissue culture plates and transfected with the miR-139-5p mimic or NC as described above. After propagation for 48 h, total RNA was extracted for the expression profiling analyses. The microarray profiling was performed as described in our previous work (Wang et al. 2014).

### Luciferase assay

Approximately 5000 HEK-293T cells per well were plated into 96-well plates and were cotransfected with 50 nmol/L of miR-139-5p mimic (or NC), 50 ng of the luciferase reporter, and 5 ng of the pRL-CMV Renilla luciferase reporter using 0.5 µL per well Lipofectamine 2000 (Invitrogen, USA). After 48 h incubation, the firefly and Renilla luciferase activities were quantified using a dual-luciferase reporter assay (Promega, USA).

### Western blot

Cellular proteins were extracted and separated in SDS-PAGE gels, first separated by 10% sodium dodecyl sulphate-polyacrylamide gel electrophoresis and then transferred to nitrocellulose membranes (Bio-Rad Laboratories, USA). The membranes were blocked with 5% nonfat milk and incubated with a mouse anti-AMFR polyclonal antibody at a dilution of 1:500 (Abcam, USA), a rabbit anti-activated NOTCH1 antibody (Abcam, USA), or a mouse anti-beta-actin monoclonal antibody at a dilution of 1:1000 (Sigma, USA). The membranes were subsequently incubated with a goat anti-mouse horseradish peroxidase secondary antibody (Sigma, USA). The protein complex was detected using enhanced chemiluminescence reagents (Pierce, France).

### Immunohistochemical staining

Tissue arrays were constructed using 134 paired CRC tissues and NCTs. Immunohistochemical staining was performed on 4 μm sections of paraffin-embedded tissues to determine the expression level of AMFR protein. In brief, the slides were incubated in AMFR and NOTCH1 antibody diluted 1:200 at 4°C overnight. The subsequent steps were performed using the EnVision™ FLEX High pH visualisation system according to the manufacturer’s instructions (DAKO, Demark). The scoring of AMFR and NOTCH1 was performed as previous described (Tsai et al. 2007; Skrtic et al. 2010).

### Statistical analyses

The results are presented as the mean values ± SEM. The data were subjected to Student’s *t*-tests, the Mann-Whitney *U* test or the Kruskall-Wallis test unless otherwise specified (χ^2^ test, Spearson’s correlation). The overall survival curves were plotted according to the Kaplan-Meier method, with the log-rank test applied for comparisons. A *P* value of less than 0.05 was considered statistically significant. SPSS 16.0 package (IBM, USA) and Graphpad prism 5.0 software (GraphPad Software, USA) were used for statistical analyses and scientific graphing, respectively.

## Electronic supplementary material

Below is the link to the electronic supplementary material.

Supplementary material 1 (XLSX 48 kb)

Supplementary material 2 (PDF 345 kb)

## ABBREVIATIONS

AMFR: autocrine motility factor receptor
CRC: colorectal carcinoma
miRNA: microRNA
qRT-PCR: quantitative reverse transcription polymerase chain reaction
siRNA: small interfering RNA
3′UTR: 3′ untranslated region
