# Correction to: MiR-139-5p inhibits migration and invasion of colorectal cancer by downregulating AMFR and NOTCH1

**DOI:** 10.1007/s13238-021-00826-x

**Published:** 2021-02-09

**Authors:** Mingxu Song, Yuan Yin, Jiwei Zhang, Binbin Zhang, Zehua Bian, Chao Quan, Leyuan Zhou, Yaling Hu, Qifeng Wang, Shujuan Ni, Bojian Fei, Weili Wang, Xiang Du, Dong Hua, Zhaohui Huang

**Affiliations:** 1 Wuxi Oncology Institute, the Affiliated Hospital of Jiangnan University, Wuxi, 214062 China; 2 Department of Surgical Oncology, the Affiliated Hospital of Jiangnan University, Wuxi, 214062 China; 3 Department of Radiation Oncology, the Affiliated Hospital of Jiangnan University, Wuxi, 214062 China; 4 Department of Pathology, Fudan University Shanghai Cancer Center, Shanghai, 200032 China

## Correction to: Protein Cell (2014) 5(11):851–861 10.1007/s13238-014-0093-5

In the original publication the display of Fig. 1 is incorrect. The correct Fig. 1 is available in this correction.

**Figure 1:**
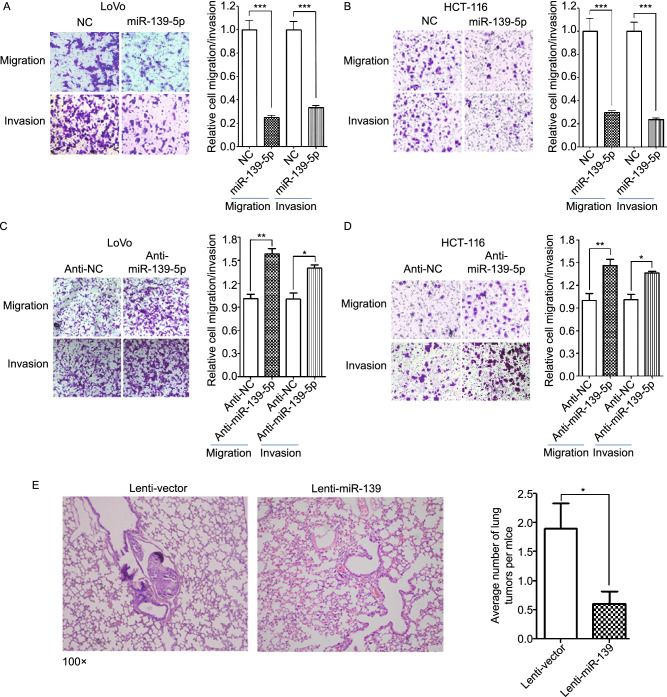
**miR-139-5p is frequently downregulated and associated with poor overall survival in CRC.** (A) MiR-139-5p expression was detected by quantitative reverse transcription polymerase chain reaction (qRT-PCR) in 80 paired CRC and adjacent noncancerous tissues (NCTs). MiR-139-5p expression was markedly downregulated in tumor tissues compared with the corresponding NCTs (U6 small nuclear RNA was used as an internal control). (B) Overall survival analysis based on the expression level of miR-139-5p. MiR-139-5p expression was examined in 158 CRC tissues, and these cases were divided into two groups (high or low) or four groups (1–4) based on their miR-139-5p levels in tumors. MiR-139-5p expression was positively correlated with the overall survival

