# Supplementary material for: MiR-139-5p inhibits migration and invasion of colorectal cancer by downregulating AMFR and NOTCH1
Source: Protein Cell. 2014 Aug 23;5(11):851–61. doi: 10.1007/s13238-014-0093-5 (PMC4225484; doi:10.1007/s13238-014-0093-5)
Supplement: Supplementary file 2 — Supplementary material 2 (PDF 345 kb) [file 13238_2014_93_MOESM2_ESM.pdf]

Supplementary Figures Legends and Tables

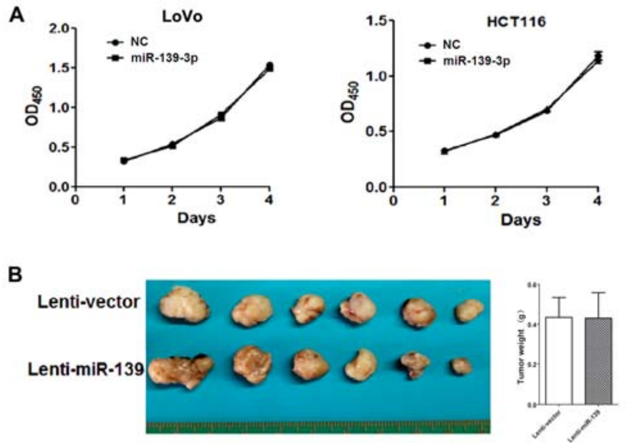

Figure S1. miR-139-5p showed no significant effect on CRC cell growth. (A) miR-139-5p overexpression showed no significant effect on the cell proliferation in LoVo and HCT-116 cells. (B) The effect of miR-139 on tumor formation in a nude mouse xenograft model. LoVo cells (2×10^6) stably expressing miR-139 or the control were injected s.c. into the right flank of each nude mouse.

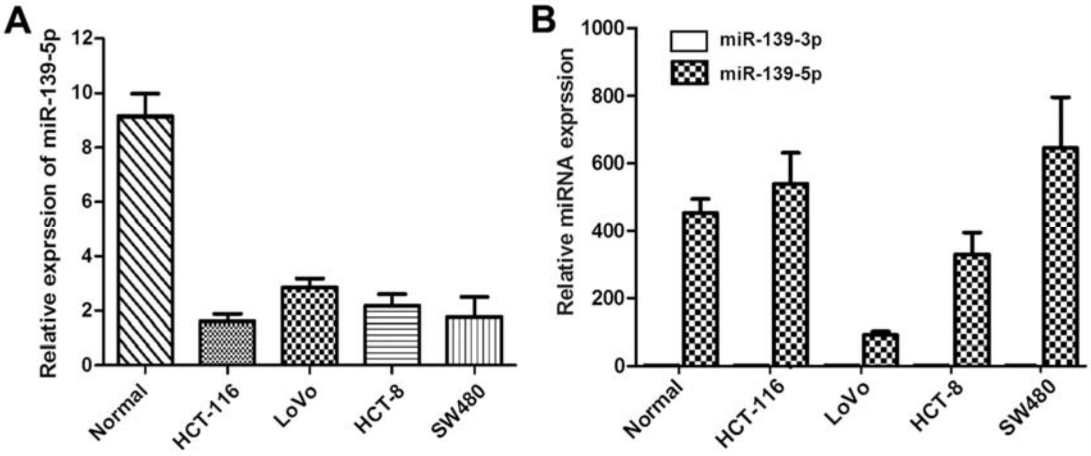

Figure S2. Expression of miR-139 in CRC cells. (A) The expression of miR-139-5p in CRC cells and tissues. Five samples of normal colon epithelium mucosae were pooled and subjected to quantitation of miR-139-5p expression (Normal). (**B)** The relative expression of miR-139-3p in 4 different CRC cells and normal tissue was much lower than that of miR-139-5p. The expression of miR-139-3p was set as 1.

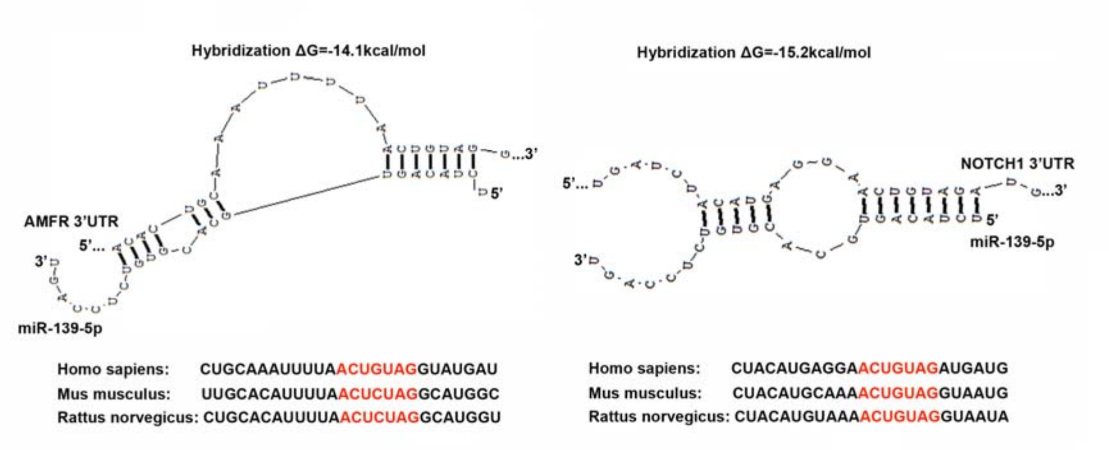

Figure S3. The predicted binding site of miR-139-5p in the 3'UTRs of AMFR and NOTCH1 is conserved among mammals.

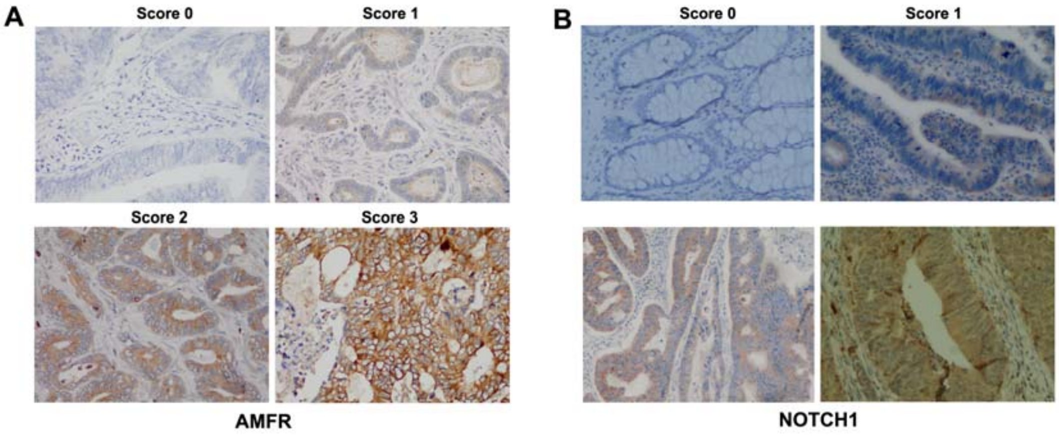

Figure S4. Immunohistochemical staining of AMFR and NOTCH1 in the CRC and NCT samples. The scores (0, 1, 2 and 3) are based on the intensity of the brown staining.

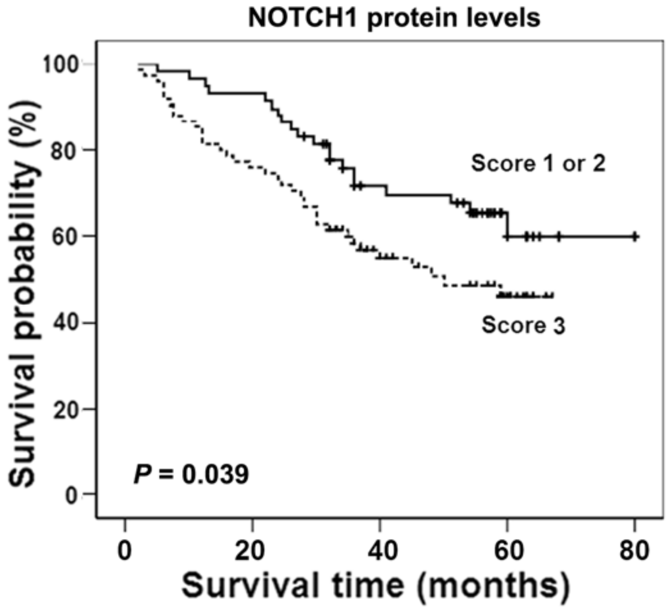

Figure S5. Survival analysis based on the NOTCH1 protein expression. The groups were ranked according to the NOTCH1 staining intensity.

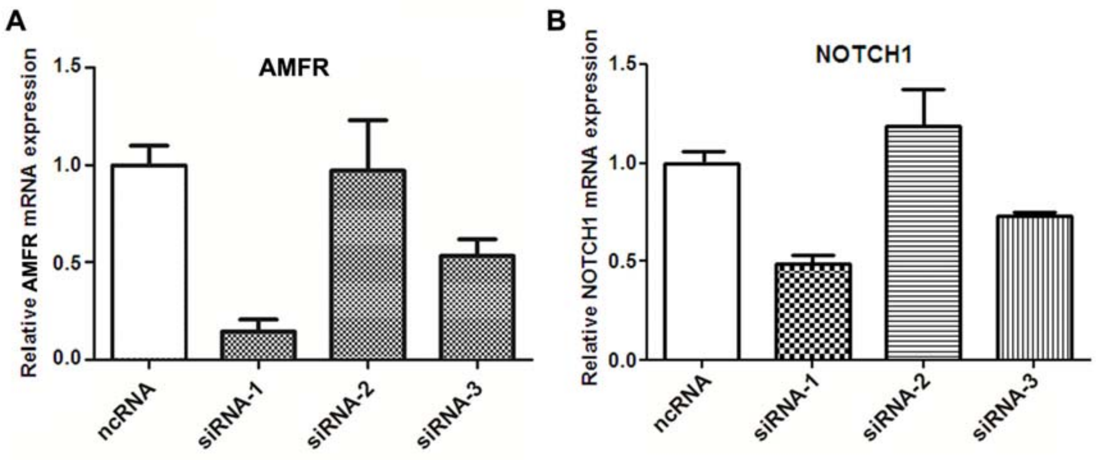

Figure S6. The knockdown of AMFR and NOTCH1 in CRC cells. The mRNA levels of AMFR and NOTCH1 were determined by qRT-PCR in LoVo cells transfected with si-AMFR, si-NOTCH1, or si-control (ncRNA). Beta-actin served as an internal control. The most effective siRNA (1#) was selected out for further investigation.

Table S1. Cox multivariate analyses for overall survival

Variables	Overall survival		*P* value
	HR	95%CI	
Age (≥58 *vs.*＜58)	1.044	0.616-1.771	0.873
Gender (female *vs.* male)	0.587	0.341-1.011	0.055
Tumor size(⩾5cm *vs.* <5)	1.031	0.610-1.743	0.908
Grading (G3 *vs.* G1-2*)*	1.927	1.080-3.440	0.026
TNM stage(III+IV *vs.* I+II)	4.225	2.861-6.239	<0.0001
miR-139-5p levels(high *vs.* low)	2.681	1.532-4.691	0.001

HR:hazard ratio; CI:confidence interval; TNM: tumor-node-metastasis classifications.

Table S2. Lung metastases in the mouse models of pulmonary metastasis

	Lung metastases	
No.	Lenti-vector	Lenti-miR-139
1	2	0
2	1	1
3	1	0
4	0	1
5	2	0
6	5	0
7	2	2
8	2	1
9	3	0
10	1	1
Total	19	6

Table S4. Patients' information

Characteristics	Colorectal cancer* （n=158）	Colorectal cancer# （n=134）
Gender		
Male	93	74
Female	65	60
Age at diagnosis		
⩾58	80	69
<58	78	65
TNM stage		
I	22	18
II	48	39
III	67	58
IV	21	19
Nodal status		
Positive	86	45
Negative	72	38
Tumor size (cm)		
<5	96	84
⩾5	62	50
Tumor location		
Rectum	72	57
Distal colon	37	34
Proximal colon	49	43
Grading		
G1	6	6
G2	124	105
G3	28	23

*: Of these 158 cases, matched frozen noncancerous tissues were available in 80 cases.

#: Of these 158 cases, the data of miR-139-5p, AMFR and NOTCH1 expression were available in 134 cases.

Table S5. Primer sequences

Primers	Sequence (5 'to 3')	Products length
**Primers for pri-miR-139 cloning**		
	GGTGAGGGACTGAGGTGAT	890
	CAGGGTTTCTGATACAGTAGGT	
miR-139-5p-F	CGACGCGTCCCTCTTCCCATTCCTTC	756
miR-139-5p-R	CCGGAATTCCGAGACCCACTGACACTATCT	
**Real time PCR Primers**		
AMFR-F	TCCAAGGCAGGTAGGTTCA	259
AMFR-R	GGAGTGGTTAGGCAGCAAG	
NOTCH1-F	GCAGTCAGGCGTGTTGTTC	147
NOTCH1-R	GGCACTTTCTGTGAGGAGGA	
β-actin-F	AGTGTGACGTGGACATCCGCAAAG	220
β-actin-R	ATCCACATCTGCTGGAAGGTGGAC	
**3'UTR primer**		
LAPTM4B-Out-F	ATACGGCAACTGCCTCCTA	883
LAPTM4B-Out-R	CTGGTGCTTTCTAATGGTCTT	
LAPTM4B-In-F	GGGGTACCAACTCCTCTGATGTCCTGGTT	528
LAPTM4B-In-R	CGGGATCCCTATGCTGGAATGGCTGAA	
HNRNPF-Out-F	CTTCAGTGTTTTCTCATGCAA	863
HNRNPF-Out-R	TCACCATTTCCACCATTCA	
HNRNPF-In-F	CGGAATTCTCTTTTACACCACATCACAG	630
HNRNPF-In-R	CGGGATCC ACTTTTATTTAGCCTCATCA	
TOP1-Out-F	GCTACTGTATGCAAAGT	852
TOP1-Out-R	CCATTAAGTTGTAGGAA	
TOP1-In-F	GGGGTACCCCCTAATCTTTCACTTG	341
TOP1-In-R	CGGGATCCCATTAAGTTGTAGGAATT	
AMFR-Out-F	CAGCGTAAGGACGAACTCC	1536
AMFR-Out-R	TTCCACAACAACGACAGCA	
AMFR-In-F	CGGAATTCTGGTCTTATAGTGTTTGGACA	1277
AMFR-In-R	CGGGATCCCAACGACAGCAGTTTGATA	
AMFR-M-F	CGGAATTCTGGTCTTAGCACTTTTGGACA	204
AMFR-M-R	CGGGATCCCAACGACAGCAGTTTGATA	196
NOTCH1-Out-F	GTTCTTGAAATGTAGGCATCA	900
NOTCH1-Out-R	CTCCCTCAGAGCATAGCAG	
NOTCH1-In-F	CGGAATTCTAGGAGACTTGCCAGAGCC	591
NOTCH1-In-R	CGGGATCCTGGAAGCCAGATCACCATC	
NOTCH1-M-F	CGGAATTCTAGGACATTTGCCAGAGCC	198
NOTCH1-M-R	CGGGATCCTGGAAGCCAGATCACCATC	121
**Primers for the cloning of AMFR ORF**		
AMFR-ORF-Out-F	CCTGGGGGCCAGAGGTC	2221
AMFR-ORF-Out-R	ATGCAGGAGCACCAGAGTTC	

AMFR-ORF-In-F	ATAGGATCCCCGCCATGCCGCTGCTCTTC	2031
AMFR-ORF-In-R	ATGGAACTAGTAGAATTGGGACAGGCCTCC TCCAGTCAG	
